# A surgical case of solitary severe tricuspid regurgitation mimicking constrictive pericarditis

**DOI:** 10.1186/s44215-022-00022-1

**Published:** 2023-03-15

**Authors:** Daisuke Kaku, Hidekazu Hirai, Hiroyuki Seo, Tadahiro Murakami

**Affiliations:** Department of Cardiovascular Surgery, Saiseikai Noe Hospital, 1-3-25 Furuichi, Joto-ku, Osaka, 536-0001 Japan

**Keywords:** Tricuspid regurgitation, Constrictive pericarditis, Hemodynamics, Heart valve prosthesis implantation

## Abstract

**Background:**

It has been reported that severe tricuspid regurgitation may demonstrate constrictive pericarditis-like hemodynamics. In this article, we report a surgical case of solitary severe tricuspid regurgitation in which the hemodynamic features are consistent with constrictive pericarditis.

**Case presentation:**

The patient was a 78-year-old man with chronic atrial fibrillation and tricuspid regurgitation. He was admitted to our hospital with complaints of edema and dyspnea, and transthoracic echocardiography showed severe tricuspid regurgitation and enlargement of the bilateral atrium. A right heart catheterization revealed “dip and plateau” patterns in the biventricular pressure waveforms, and both right and left ventricle endo-diastolic pressure had increased to 30 mmHg. Although there were no signs of calcification or thickening of the pericardium in computed tomography, we diagnosed the patient with constrictive pericarditis with severe tricuspid regurgitation and underwent surgical intervention since his heart failure symptoms were resistant to medication. The surgical findings did not show any pericardial thickening or adhesions either; therefore, we diagnosed the patient with solitary severe tricuspid regurgitation showing constrictive pericarditis-like hemodynamics. We performed tricuspid valve replacement with a bioprosthetic valve and his heart failure symptoms improved postoperatively.

**Conclusions:**

To determine the best timing for surgery, it is essential to recognize the existence of severe tricuspid regurgitation mimicking constrictive pericarditis.

## Background

Previous studies have demonstrated that severe tricuspid regurgitation (TR) could mimic some hemodynamic findings of constrictive pericarditis (CP). If the pericardium is intact, the expansion and increase in pressure of the right heart system due to severe TR probably increases the pressure in the pericardial cavity as well, which in turn compresses the left ventricle. Here, we report a case of a patient with solitary severe TR mimicking CP, who required surgical intervention.

## Case presentation

A 78-year-old Japanese man with chronic atrial fibrillation and TR was admitted to our hospital because of dyspnea and worsening systemic edema. His weight had increased by around 7 to 70.0kg. He had been receiving outpatient treatment with diuretics for 6 years. He had no history of cardiac surgery, radiation therapy, tuberculosis, rheumatic fever, or chest trauma. On admission, his vital signs were normal, but oxygen saturation had decreased to 93% on room air. Physical examination revealed jugular vein distension, a pan-systolic murmur (Levine 2/6) at the 3rd intercostal space at the left sternal border, and moderate lower limb edema. The liver was palpable 3 fingerbreadths below the costal margin.

**Fig. 1:**
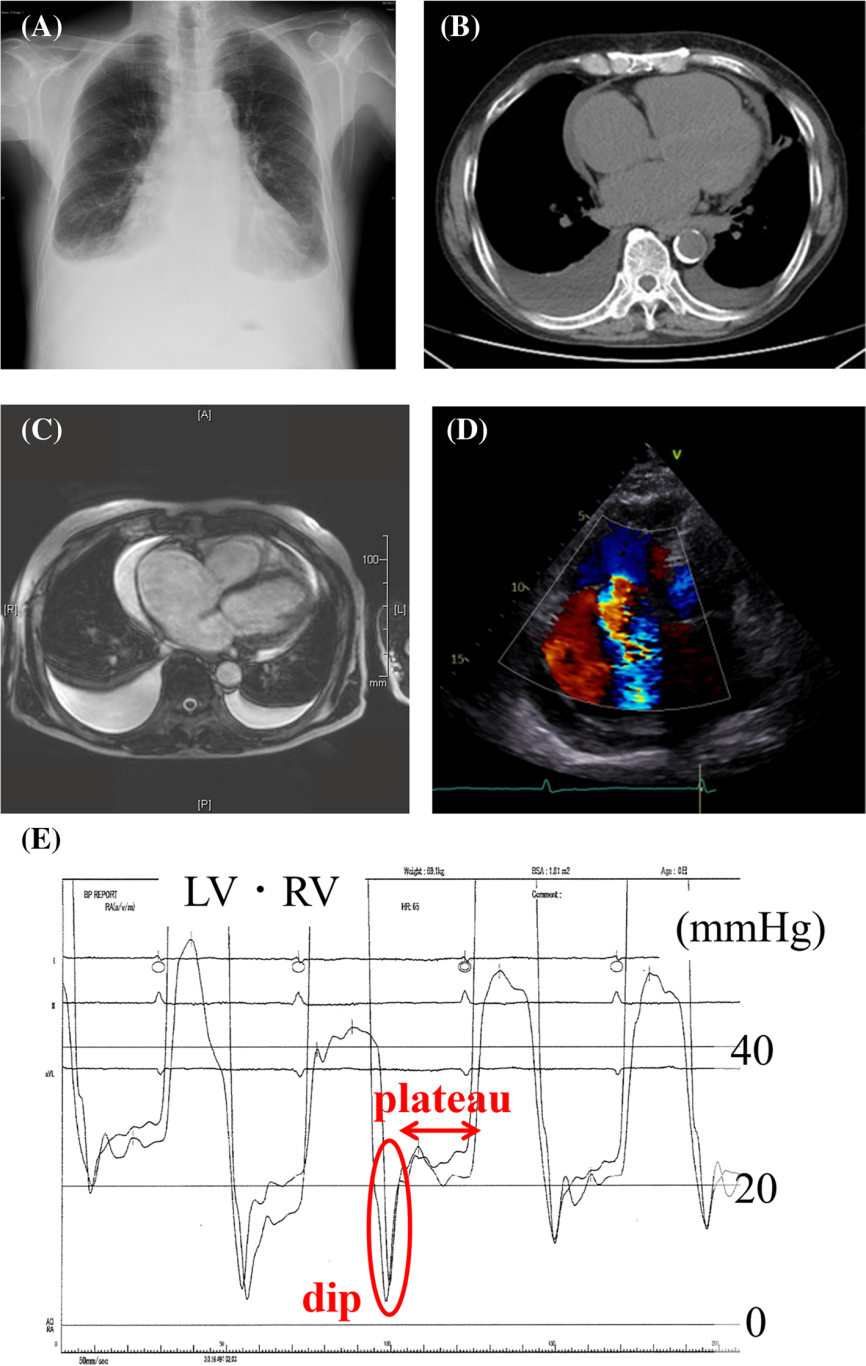
**A** Chest X-ray showed cardiomegaly and bilateral pleural effusion. No calcification of the pericardium was observed. **B** Bilateral pleural effusion and a small amount of pericardial effusion were detected in computed tomography. No calcification or thickening of the pericardium was observed. **C** Cardiac magnetic resonance imaging (cine image) demonstrated no thickening of the pericardium, nor adhesion between myocardium and pericardium. **D** Trans-thoracic echocardiographic color Doppler image of apical four-chamber view showed solitary severe TR and enlarged bilateral atria. **E** The pressure waveforms obtained for both the right and left ventricles on right heart catheterization demonstrated the “dip and plateau” pattern typical of constrictive pericarditis

We observed mild anemia and renal dysfunction in the patient on laboratory investigations. Chest X-ray and computed tomography showed cardiomegaly and bilateral pleural effusion, although we did not observe calcification or thickening of the pericardium (Fig. 1A, B). Cardiac magnetic resonance imaging also demonstrated no thickening of the pericardium, nor any adhesion between the myocardium and pericardium (Fig. 1C).

Trans-thoracic echocardiography revealed solitary severe TR and enlarged bilateral atria (Fig. 1 D). The respiration-related ventricular septal shift was not significant and the respiratory changes in the trans-mitral and trans-tricuspid flow patterns were 30% and 18%, respectively.

On right heart catheterization, the mean right atrial pressure was 30 mmHg, which was almost identical to the right and left ventricular end-diastolic pressures and the mean pulmonary capillary wedge pressure, indicating the equalization of diastolic pressures in all cardiac chambers (Table 1). The pressure waveforms of both ventricles demonstrated the “dip and plateau” pattern typical of CP (Fig. 1E).

**Table 1:** Improvement in the parameters measured by right heart catheterization after surgery

	Parameters (mmHg)				
	PCWP; systolic/diastolic (mean)	PAP; systolic/diastolic (mean)	RAP; systolic/diastolic (mean)	RVP; systolic/diastolic (end-diastolic)	LVP; systolic/diastolic (end-diastolic)
Preoperative^*^	48/29 (31)	51/24 (34)	47/23 (30)	46/12 (21)	140/13 (25)
Postoperative		21/5 (11)^a^	29/16 (20)	32/18 (18)	

*Preoperative data indicates the equalization of diastolic pressures in all cardiac chambers

^a^PAP improved significantly after surgery. We collected postoperative data the day after surgery

*PCWP* pulmonary capillary wedge pressure, *PAP* pulmonary artery pressure, *RAP* right atrial pressure, *RVP* right ventricular pressure, *LVP* left ventricular pressure

Despite receiving medication for heart failure, along with diuretics including tolvaptan (15mg/day), azosemide (30mg/day), torsemide (8mg/day), and human atrial natriuretic peptide (0.025γ), his weight only decreased to 65.2kg (still 2kg more than normal) and dyspnea requiring oxygen administration remained. Therefore, although only the “dip and plateau” pattern in right heart catheterization data met the diagnostic criteria of CP, we diagnosed the patient with CP and severe TR because of his unresponsiveness to medication and proceeded to surgery.

We performed the operation via median sternotomy, under cardiopulmonary bypass and cardiac arrest. Intraoperatively, the mean pulmonary artery pressure did not decrease but remained between 35 and 38 mmHg when we opened the pericardium. There were no pericardial adhesions in the pericardial sac, nor any gross pericardial thickening. The tricuspid valve annulus was enlarged to 4fingerbreadths, the anterior leaflet was thickened and decreased mobility, and the posterior and septal leaflets were extremely shortened; therefore, we are concerned that tricuspid valve annuloplasty alone might be inadequate to control his severe TR and performed tricuspid valve replacement with a Carpentier-Edwards Perimount mitral valve bioprosthesis (Edwards Lifesciences, Irvine, CA). The time of operation, cardiopulmonary bypass, and cross-clamp were 235, 105, and 73 min, respectively. The mean pulmonary artery pressure improved markedly, from 38 to 21 mmHg, after tricuspid valve replacement (Table 1).

Postoperatively, the patient’s body weight decreased to 53.5kg (16.5 and 11.8kg weight loss compared to admission and preoperation, respectively), his dyspnea and systemic edema improved, and there was no TR on echocardiography. The administration of diuretics for heart failure was reduced to furosemide (40mg/day) only, and he was discharged from the hospital about 1 month after the operation. He is currently undergoing outpatient follow-up and has not experienced any valve-related events. Therefore, we finally considered that his CP-like hemodynamics were caused by solitary severe TR because of the control of severe TR by tricuspid valve replacement brought about a decrease in pulmonary artery pressure and improvement of right heart failure.

## Discussion and conclusions

We experienced a rare surgical case of solitary TR exhibiting CP-like hemodynamics. We planned surgery for TR and CP preoperatively, but found neither adhesion nor thickening intraoperatively. The surgeons experienced difficulty determining intraoperatively whether treatment for TR alone would be sufficient. Therefore, TR mimicking CP hemodynamics should also be considered preoperatively.

Several cases of solitary TR showing similar hemodynamics have previously been reported [1, 2]. To explain its pathophysiology, one report suggested the concept of pericardial constraint [3], which states that an increase in pressure in the right heart system due to TR increases the pressure in the pericardial cavity (a closed cavity), which leads to an increase in pressure in the left heart system as well. Similarly, an increase in pressure in the right heart system was observed by Swan-Ganz catheterization in our case; therefore, we believe that CP-like hemodynamics were caused by pericardial constraint.

Although the indications for surgery for TR differ depending on whether TR is primary or secondary, surgery is indicated for patients in whom right heart failure is poorly controlled despite medical treatment. Wang et al. reported that surgery for solitary TR, especially tricuspid repair when feasible, achieved good results when it was performed before the recurrence of right heart failure, that is, before the patient develops the conventional indications for surgery [4]. Our patient had secondary solitary TR due to atrial fibrillation and presented with right heart failure refractory to medical treatment; therefore, we considered early surgical intervention to be appropriate. However, in the selection of the surgical procedure, we should have attempted tricuspid valve repair before replacement.

Echocardiography and right heart catheterization are performed to evaluate right heart failure preoperatively. Studies show that the right ventricular end-systolic area > 20 cm^2^ by echocardiographic measurement could be a predictor of surgical outcome for solitary TR [5]. Our patient’s echocardiography findings matched this criterion; therefore, we assumed that the patient would have a good postoperative outcome and decided to perform surgery, which proved successful.

In conclusion, it is important to keep in mind that solitary severe TR can mimic CP when deciding on an optimal timing for surgical intervention for TR.

## Data Availability

The datasets used and/or analyzed during the current study are available from the corresponding author on reasonable request.

## Abbreviations

TR: Tricuspid regurgitation
CP: Constrictive pericarditis
